# Mechanisms of Immune Control of Mucosal HSV Infection: A Guide to Rational Vaccine Design

**DOI:** 10.3389/fimmu.2019.00373

**Published:** 2019-03-06

**Authors:** Naomi R. Truong, Jacinta B. Smith, Kerrie J. Sandgren, Anthony L. Cunningham

**Affiliations:** ^1^Centre for Virus Research, The Westmead Institute for Medical Research, Sydney, NSW, Australia; ^2^Sydney Medical School, The University of Sydney, Sydney, NSW, Australia

**Keywords:** vaccine development, herpes simplex, antibody, T cells, adjuvants, innate immunity

## Abstract

Herpes Simplex Virus (HSV) is a highly prevalent sexually transmitted infection that aside from causing cold sores and genital lesions, causes complications in the immunocompromised and has facilitated a large proportion of HIV acquisition globally. Despite decades of research, there is no prophylactic HSV vaccine ready for use in humans, leaving many questioning whether a prophylactic vaccine is an achievable goal. A previous HSV vaccine trial did have partial success in decreasing acquisition of HSV2–promising evidence that vaccines can prevent acquisition. However, there is still an incomplete understanding of the immune response pathways elicited by HSV after initial mucosal infection and how best to replicate these responses with a vaccine, such that acquisition and colonization of the dorsal root ganglia could be prevented. Another factor to consider in the rational design of an HSV vaccine is adjuvant choice. Understanding the immune responses elicited by different adjuvants and whether lasting humoral and cell-mediated responses are induced is important, especially when studies of past trial vaccines found that a sufficiently protective cell-mediated response was lacking. In this review, we discuss what is known of the immune control involved in initial herpes lesions and reactivation, including the importance of CD4 and CD8 T cells, and the interplay between innate and adaptive immunity in response to primary infection, specifically focusing on the viral relay involved. Additionally, a summary of previous and current vaccine trials, including the components used, immune responses elicited and the feasibility of prophylactic vaccines looking forward, will also be discussed.

## 1. Introduction

### 1.1. The Need for a Herpes Simplex Virus Vaccine

A prophylactic vaccine for herpes simplex virus types 1 and 2 (HSV1 and 2) is a global public health priority for development, as stated by WHO (1, 2) for several reasons: (1) genital herpes caused by HSV1 or 2 is now the commonest sexually transmitted infection; (2) it causes severe disease in neonates; (3) HSV1 is the leading cause of infectious blindness in western countries; (4) prior HSV2 infection leads to a three to six fold increased risk of HIV infection globally (3–5). Up to 50% of HIV transmissions in sub-Saharan Africa are estimated to occur in a setting of HSV2 infection (6, 7) and are more likely to occur soon after HSV2 acquisition (8). Antiviral therapy for recurrent genital herpes markedly reduces clinical episodes but does not completely suppress viral shedding and does not reduce HIV acquisition (9), probably because of inadequate pharmacokinetics of acyclovir/valaciclovir (10). In contrast a prophylactic HSV vaccine would be likely to reduce HIV spread (11).

### 1.2. The History of Herpesvirus and HSV Vaccine Development

The development of vaccines for herpesviruses has met with variable success. The only major human successes have been with the vaccines for chicken pox and herpes zoster (shingles), both caused by varicella zoster virus (VZV). Progress with vaccines for herpes simplex virus has been very slow and partial so far.

Both VZV and HSV are alphaherpesviruses and their pathogenesis is similar as both infect skin and nerves and develop latent infection in trigeminal and dorsal root ganglia (TG and DRG), from where they reactivate, but much more frequently for HSV. The live attenuated varicella virus Oka strain (Varivax) was shown to prevent chicken pox in Japan in the 1990s and has been successfully deployed worldwide. Then in 2005, “Zostavax,” consisting of a 14-fold more concentrated preparation of the Oka strain, was shown to prevent herpes zoster in 51% of immunized subjects and prolonged pain or postherpetic neuralgia (PHN) in 65% of them. However, vaccine efficacy against the incidence of zoster, although not PHN, is diminished in subjects >70 years of age and markedly declines over 8 years (12, 13).

Unlike live attenuated vaccines, recombinant protein vaccines require combination with an adjuvant to stimulate the immune system. Adjuvants enhance the immune response to an antigen and direct the immune system toward particular arms of the immune response, for example, toward T cell or antibody responses or both. This is usually mediated through antigen presenting cells, particularly dendritic cells (DCs). Recently, a recombinant protein vaccine for herpes zoster (RZV) was shown to be highly effective with >90% efficacy, even in subjects>80 years of age. There was no significant decline in protection over 4 years, with immunogenicity retained for 9 years (14). The vaccine contains a single varicella glycoprotein and the adjuvant system, AS01B, which consists of deacylated monophosphoryl lipid A (dMPL) and QS21, formulated in liposomes. dMPL, a toll-like receptor 4 agonist, is extracted from the cell wall of the bacterium *Salmonella Minnesota* and the saponin QS21 is derived from the bark of the soap bark tree (*Quillaja saponaria*). This adjuvant system stimulates VZV glycoprotein-specific CD4 T cells (and low level CD8 memory T cells) and humoral responses, although primary or naïve CD8 T cells are not stimulated (15).

Thus, very high levels of protection can be induced against herpes zoster by a single recombinant viral protein combined with an adjuvant that induces the appropriate adaptive (T and B cell) immune response by targeting antigen presenting cells. This is a strong improvement over the response induced by the live attenuated vaccine (16).

During 60 years of mostly unsuccessful attempts at development of an HSV vaccine, live attenuated candidates have been avoided because of concerns about potential carcinogenicity (initially as HSV2 was thought to cause cervical cancer) and recombination with clinical strains to produce new, highly virulent strains. However, new live attenuated candidates have been specifically mutated to achieve attenuation, e.g., via deletions of two key proteins, rather than simple point mutations to reduce the likelihood of reversion to virulence, and are currently in clinical trials, such as HSV529 (17). Other vaccine candidates have included DNA vaccines, hybrid recombinant viruses, and recombinant viral proteins.

In the 1990s two recombinant viral protein vaccine candidates were trialled. The Chiron vaccine candidate consisted of HSV2 entry glycoproteins B and D combined with oil in water emulsion adjuvant, MF59. When administered to subjects with recurrent genital herpes it induced high levels of neutralizing antibody but had no persistent or significant effect on frequency of recurrences (18). The GSK vaccine candidate, Simplirix, consisted of just the HSV2 entry glycoprotein D (gD), and the adjuvant system AS04. HSV2 gD is widely recognized by human populations, inducing both neutralizing antibody and CD4 T cells (19). AS04 consists of alum and dMPL. Simplirix showed 74% efficacy but only in HSV1/2 seronegative women with long-term HSV2-infected partners (20). However, the subsequent Herpevac trial of Simplirix in randomly selected HSV1 and 2 seronegative women surprisingly showed significant efficacy against genital herpes caused by HSV1 (58%) but not HSV2 (only 20% and insignificant efficacy) (21). Thus, cross-protection against HSV1 was achieved with recombinant HSV2 gD, which is highly conserved between the two serotypes (22). This protection correlated with HSV1 neutralizing antibody titers whereas HSV2 neutralizing antibody titers were low. The better efficacy of the first trial could be explained by subclinical genital exposure to HSV2 shed by the infected partner, priming a later successful vaccine response. The efficacy of the novel adjuvant dMPL was attributed to induction of CD4 Th1 T cells as well as neutralizing antibody. However, no specific CD8 T cells were induced (23).

### 1.3. What Can be Learned From Comparison of the Efficacy and Immunogenicity of the Recent Herpes Zoster and Herpes Simplex Vaccines?

Why is there such a marked difference between the remarkable efficacy of the RZV vaccine and the partial success of the Simplirix vaccine given that they are similarly formulated and how can this inform development of a better HSV vaccine? The answer probably lies in understanding the mechanisms of immune control of natural herpes zoster and initial genital herpes. These include (1) “immunotherapy” vs. prophylaxis–the vaccine for herpes zoster seeks to control a reactivation disease, whereas the HSV vaccine seeks to control primary infection and disease (2) possible differences in the immune responses required for control, (3) differences in the mechanism of action of the adjuvants and (4) immune evasion strategies of each virus. The latter is reviewed in Abendroth et al. (24) and Su et al. (25).

The distinction between an immunotherapeutic vaccine and prophylactic vaccine is critical. Prophylactic vaccines (such as Simplirix) aim at preventing acquisition of a pathogen and need to stimulate broad and durable immunity at the site of the entry of the pathogen. The Simplirix vaccine stimulated both antibody titers and CD4 T cell responses but antibody correlated best with vaccine efficacy in the Herpevac trial (23). Examination of the immune responses to HSV in nerve ganglia and skin suggest both are important, as well as CD8 T cells. Correlation of immune effectors with vaccine efficacy in trials of candidate immunotherapeutic vaccines from Agenus and Genocea as discussed below, also suggest all three are important–and perhaps are also important for prophylactic HSV vaccines. For a prophylactic vaccine to successfully stimulate the desired antibody, CD4 and CD8 T cell responses, it will need to stimulate dendritic cells (DCs), which are the only immune cell that can stimulate naïve responses.

In contrast, therapeutic vaccines aim to minimize disease severity and duration or reduce recurrences. Herpes zoster is caused by VZV reactivation in the neuronal ganglia so the RZV vaccine is effectively an immunotherapeutic vaccine. It is not intended as and may never be used as a primary prophylactic vaccine although it may effectively protect those previously immunized with Varivax from HZ when this cohort begins to reach the age of 50. This is suggested by the fact that CD4 T cell immunity generated by RZV was unaffected by previous Zostavax administration (26). RZV was demonstrated to activate blood memory T cells into a long-lasting polyfunctional state (27) which may partly explain its increased efficacy compared to Simplirix, although the presence of such T cells in critical tissues–neuronal ganglia or skin/mucosa, is unknown.

From the above discussion it is clear that definition of the innate immune response, in particular the role of critical subsets of DCs, which leads to the required effector response for prevention of infection or disease should make a major contribution to improving vaccine design. To do this it is important to know (1) the type of effector immune responses required (e.g., CD4 and/or CD8 T cells; which cytokines) and especially which pathogen proteins are most immunogenic in this setting; (2) which DCs need to be targeted to stimulate this response and their location; (3) how to target and activate those DCs with adjuvants and (4) how and where these adjuvants work; (5) any “off-target” effects of adjuvants likely to lead to unacceptable toxicity.

## 2. Immune Control of HSV

### 2.1. Innate Immunity

#### 2.1.1. Keratinocytes

Keratinocytes are the first line of defense against HSV infection in the skin and form a formidable barrier to pathogen entry. Keratinocytes also play a key role in innate immunity against pathogens (28). They express many pattern recognition receptors including Toll-like receptors (TLRs), Nod-like receptors (NLRs) and RIG-I-like receptors (RLRs) for the detection of bacterial, fungal, and viral components (29). Keratinocytes produce a vast array of antimicrobial peptides such as LL-37, β-defensins, RNases, and S100 family members (29). Additionally, keratinocytes produce chemokines and cytokines in response to pathogenic stimuli, including the chemokines CCL3, 4, and 5 in response to HSV infection. CCL3 was highly chemotactic for activated CD8 T cells, CCL4 for activated CD4 T cells, and CCL5 for resting and activated CD4 or CD8 T cells (30). Keratinocytes produce pro-inflammatory cytokines such as TNF, IL-1α, IL-1β, IL-6, IL-10, IL-18, and IL-33, which direct the immune response toward a Th1 responses, Th2/Treg responses or have direct antiviral effects (30–32). In addition, keratinocytes have also been shown to be an accessory or “non-professional” antigen presenting cell that upregulate MHC class II in response to IFN-γ produced by T cells (33, 34). In an *in vitro* model of a recurrent herpes simplex lesion, IFN-γ stimulated, HLA-DR expressing human keratinocytes were capable of both presenting HSV antigen to T cells and acting as targets for HSV-specific T cell cytotoxicity (33).

#### 2.1.2. Type I Interferon, Plasmacytoid DCs, and AXL+SIGLEC6+ DCs

Type I Interferons (IFNs) are a key component of innate antiviral immunity. They are produced by antigen presenting cells following detection of a pathogen and activation of pattern recognition receptor signaling, such as the TLR signaling pathway. The Type I IFNs expressed in humans include IFN-α (of which multiple subtypes have been identified), IFN-β, IFN-ε, IFN-ω, and IFN-κ, although the functions of IFN-α and -β have been best characterized (35, 36). Type I IFNs induce the expression of antiviral genes known as IFN stimulated genes (ISGs), which play a role in inhibiting viral replication and promoting degradation of viral mRNA (36). Type I IFNs also activate multiple immune cell types in response to HSV infection, including neutrophils, macrophages, natural killer cells, and DCs (35).

Plasmacytoid dendritic cells (pDC) are extremely potent producers of IFN-α, and thus play an important role in antiviral defense. pDCs can also produce other cytokines and chemokines such as TNF, IL-6, CXCL10, and CCL3, for the recruitment and activation of other immune cells (37). Additionally, pDCs are thought to contribute to adaptive immunity through the activation of T cells. Viral stimulation not only triggers IFN-α, but can also differentiate pDCs into antigen presenting cells, via the upregulation of HLA-DR, CD80, and CD86, that are capable of T cell stimulation and cytokine production (38). In particular, studies of both mouse and human pDCs have demonstrated cross-presentation of exogenous antigens, resulting in the activation of naïve or memory CD8 T cells (39, 40).

In a study of human recurrent genital herpes lesions, pDCs infiltrated at both early (day 4) and late (day 10) phases. They were often found at the dermo-epidermal junction and were closely associated with CD69^+^ T cells as well as NK cells (41). Despite expressing the HSV entry receptors nectin1, nectin2, and HVEM, pDCs were resistant to HSV infection *in vitro*, but were able to stimulate virus-specific autologous T cell proliferation, particularly in CD8 T cells, indicating their capacity to cross-present antigens. This study demonstrated specifically in the context of HSV that pDCs are both strong producers of IFN-α and stimulated T cell proliferation in response to the virus. However, more recent studies challenge the notion that T cells proliferation is stimulated by pDCs.

Recently, a new DC sub population with characteristics of both conventional (c)DC and pDC has been described. AS DCs, named for their expression of AXL and SIGLEC6, express markers in common with both pDCs and cDCs (42). Upon cell sorting to obtain pure populations, it was found that pDCs were the producers of Type I IFN with weak ability to stimulate T cell proliferation, while AS DCs had the inverse functional responses. This study also provides evidence that traditional pDC gating is contaminated with AS DCs, suggesting that previous work investigating the role of pDCs in HSV infection would also contain contaminating AS DCs, and that AS DCs may be the true stimulators of T cell proliferation.

No work has as yet been conducted on AS DCs in relation to HSV infection, and it has not been assessed whether they are recruited to the skin during inflammation, as has been shown for pDCs. Therefore, studies investigating the presence of AS DCs in the HSV inflammatory infiltrate need to be conducted. By establishing what role AS DCs play in response HSV infection, it may bifurcate functional roles previously thought to be carried out by pDCs, i.e., Type I IFN and T cell stimulation.

#### 2.1.3. Natural Killer and Innate Lymphoid Cells

Several studies point to an important role for natural killer (NK) cells in response to HSV infection, particularly in controlling the severity of infection. In mouse studies, mice that lack NK cells or are depleted of NK cells have increased susceptibility to HSV2 infection and increased viral titers in the vaginal mucosa, spinal cord, and brain stem (43, 44). Similarly, a more recent study examining the severity of cutaneous HSV infection in mice with atopic dermatitis (AD) compared to normal mice found that AD mice had defective NK cell activity, which correlated with increased severity of skin infection. Furthermore, normal mice that were depleted of NK cells prior to HSV infection also had increased skin inflammation and viral titers compared to those with NK cells present (45). In humans, case studies examining patients with a specific lack of NK cells have correlated this with increased susceptibility to severe HSV infections (46, 47), suggesting an important role for NK cells in control of HSV. Furthermore, enrichment of NK cells has been observed in recurrent herpes lesions (48), interacting with pDCs (41), and CD4 T cells (49). In *in vitro* studies, TLR2-stimulated NK cells could directly activate HSV gD-specific CD4 T cells (49), and their high frequency of contact with CD4 T cells in herpetic lesions suggests they play a role in stimulating CD4 T cells in this setting. These studies indicate that NK cells play a role in controlling HSV infection by restricting viral replication and spread through the early production of IFNγ, and may also be important stimulators of adaptive immunity.However, studies in both mice and humans have not identified a correlation between NK cell activity and viral clearance, which appears to be the role of T lymphocytes (48, 50–52).

In recent years knowledge of the network of innate lymphocytes has become more complex. NK cells are part of a network of innate lymphoid cells (ILCs), whose functions are analogous to T cell subsets (53). NK cells can be considered the innate counterpart of CD8 T cells, while ILC1, ILC2, and ILC3 represent the innate counterparts of CD4 T helper 1 (Th1), Th2 and Th17 cells, identified by the same transcription factors and cytokines: NK/CD8 express Eomes, granzymes and IFN-γ, ILC1/Th1 express Tbet and IFN-γ, ILC2/Th2 express Gata-3 and IL-4, IL-5, and IL-13, and ILC3/Th17 express RORγt or AHR, IL-17, and IL-22 (53). ILCs preferentially localize into barrier tissues such as the skin, lungs and gut (54). Recently, a study examined the *in situ* ILC subset quantities and distribution in human skin (55) and found that there were differences in the proportions of different ILC subsets in normal, AD and psoriasis skin. Additionally there were increased numbers of ILCs in both AD and psoriasis compared to normal skin. However, the location of the ILC subsets was consistent: located in the upper dermis, close to the epidermis, not associated with blood vessels and in close proximity to T cells. Since ILCs were shown to infiltrate into inflamed skin, they are therefore likely to also be present in the inflammatory infiltrate during HSV infection. However, to date, no studies have investigated the presence and role of ILCs in HSV infection.

### 2.2. Adaptive Immunity

#### 2.2.1. The Role of B Cells and Neutralizing Antibodies in HSV Infection

B cells are the key immune cells of the humoral immune response, producing antibodies, such as IgG and IgA, that protect against many infectious pathogens.Levels of IgG and mucosal IgA are increased in vaginal secretions of mice, guinea pigs, and non-human primates intravaginally vaccinated with HSV2 (56–58), as well as in cervical secretions of women with primary HSV2 infection (56). Antibody responses vary, with IgG present as early as a few days while IgA presents up to 2 weeks post infection, however both persist for weeks after infection. Both antibodies react to various HSV glycoproteins, including gD, gB, and gC (56).However, the role of antibody-mediated protection against HSV2 pathogenesis is unclear (36) and data from vaccine trials is contradictory. In a human *in vitro* model of fetal dorsal root ganglia (DRG) innervating autologous epidermal skin explants, neutralizing antibodies reduced transmission of virus from axons to epidermis by 90% by binding to the virus in the intercellular gaps between axon termini and epidermal cells. It was suggested that antibodies might also be effective in preventing epidermis-to-neuron transmission during primary HSV infection (59). Some murine studies have demonstrated the importance of the antibody response to HSV. One study demonstrated that adoptive transfer of IgG from HSV2 vaccinated mice reduced viral load and pathological signs of disease in the vaginal lumen of naïve mice (60). Later studies showed that antibodies played a role in controlling viral titers and protection with the use of B cell-deficient mice (61, 62). Further studies in mice and guinea pigs have correlated pan-HSV2 antibodies with protection from vaginal challenge (63). However, other murine studies have shown that humoral immunity alone was unable to control HSV infection and failed to protect against infection. Two studies that compared T cell and B cell depletion found that T cells, rather than B cells, were critical for protection against lethal challenge of HSV2 (64, 65). Additionally, passive transfer of immune serum or anti-HSV antibodies did not protect against vaginal infection (66, 67).

However, most recently, the importance of neutralizing antibodies has once again been demonstrated in studies of a trivalent vaccine containing HSV2 gC, gD, and gE with CpG and alum in rhesus macaques. When the vaccine was administered before virus challenge it induced plasma and mucosae neutralizing antibodies that blocked gD and gE immune evasion activities and stimulated CD4 T cell responses. In guinea pigs, the trivalent group had genital lesions on <1% of days and shedding of virus on 0.2% of days (68). When the vaccine was administered to guinea pigs previously infected with HSV2, the vaccine significantly boosted ELISA and neutralizing antibody titers, reduced the frequency of recurrent lesions and vaginal shedding of HSV2 DNA by approximately 50% and almost completely prevented viral shedding (69). Therefore, neutralizing antibodies were protective against vaginal challenge and contributed significantly to reductions in genital lesions and viral shedding in animal models.

#### 2.2.2. Maternal Immunization: Clues for Protection

Studies of neonatal herpes and the protective effects of maternal immunization also provide some strong evidence for the importance of neutralizing antibodies in protection against HSV infection. Neonatal HSV infections are rare, but cause considerable morbidity and mortality in infants, with an estimated fatality rate of 60% worldwide (70).Globally, there are an estimated 14 000 cases annually, with the highest prevalences in Africa and the Americas (70). The risk of neonatal herpes infection is highest in mothers who have first-episode primary infection at the time of delivery, with transmission rates up to 60%, whereas babies born to mothers with recurrent HSV are only 1–2% likely to develop neonatal herpes (71–73). This is consistent with the hypothesis that maternal immunity provides protection to the neonate.

During pregnancy, antibodies (mostly IgG) are transferred from mother to child across the placenta (74), to ensure the temporary health and survival of the young infant. Low neutralizing antibody titre and avidity have been identified as risk factors for transmission to neonates (71, 72). However, not all pregnant women have protective concentrations of antibodies against pathogens (75), and thus maternal immunization may be an avenue for protection. Maternal immunization has already been shown to provide protection to neonates against tetanus and seasonal influenza (75) and could also be an avenue to protect against neonatal herpes.

Limited studies have investigated the effects of maternal immunization against neonatal herpes in humans, with most work conducted in mice. Murine studies have produced some promising but also conflicting results. In one study that utilized vaccination with a replication defective HSV2 mutant, HSV specific IgG antibodies passively transferred from mother to pup and reduced dissemination of virulent HSV but replication of virus or spread of virus to the CNS in pups was not prevented (76). More recently another study that utilized vaccination with a △gD-2 HSV2 showed that maternal immunization did lead to protection from neuronal involvement of HSV and latency in the pups. Increased antibody levels were also found in the serum of these pups (77). Differences in these findings could be due to the viruses the female mice were immunized with, as the more recent study used a virus that was known to protect adult mice from HSV infection upon re-challenge.

Another recent study found that both mice and humans had HSV specific antibodies in the trigeminal ganglion (TG) during HSV1 latency. Furthermore, in a murine model they demonstrated that maternal IgG accessed and persisted in neonatal TG and was protective not only against disseminated infection but also against neurological disease following neonatal HSV challenge (78). These recent studies provide evidence that maternal immunization could provide protection against neonatal herpes, and that neutralizing antibodies play a critical role in mediating this protection.

Overall, evidence suggests that humoral immunity is likely to play an early beneficial role in primary HSV infection, and may be particularly beneficial in preventing vertical transmission from mother to neonate, but ultimately cell-mediated immunity is necessary for HSV clearance and protection (36).

#### 2.2.3. The Role of T Cells in HSV Infection

CD4 and CD8 T cells are the key components of the cell-mediated immune response. CD4 T cells are critical for the activation of B cells and antibody class-switching, as well as for “licensing” DCs to activate CD8 T cells (79, 80). CD4 T cells also secrete the Type II IFN, IFN-γ, which performs a number of antiviral roles including limiting HSV viral replication and spread (81) through the induction of antiviral genes such as protein kinase RNA-activated (PKR), which inhibits translation within infected cells (82). CD8 T cells have the important role of killing virally infected cells via their cytotoxic components perforin and granzymes, mediated through the engagement of MHC class I molecules on target cells (82). HSV T cell immunity operates at two sites–neuronal ganglia and the mucosa.

In mice, CD4 and CD8 T cells surround the neurons and adherent satellite cells of trigeminal ganglia (TG) and control latency and (some) reactivation. CD8 T cells secrete granzymes which degrade intracellular ICP4 and contribute to this control (83, 84). In the human TG or DRG, there are abundant HSV infected neurons ( 3% of 27000 neurons per DRG). Effector memory CD4 and CD8 T cells expressing IFN-γ, TNF and CCL5 are found in HSV DNA^+^ ganglia and occasional clusters of these CD4 and CD8 T cells are found around neurons and are HSV specific and activated (CD69^+^). The satellite cells surrounding neurons express MHC class II, IL1 and TGF-β which can support (resident) memory T cells. Whether these T cells are truly tissue resident memory (T_*RM*_) cells has not been confirmed (85, 86).

From early studies of human recurrent herpes lesions in genital skin and mucosa, we know CD4 T cells infiltrate early and are the predominant T cell subset in the first 12–48 h post onset (52). CD4 T cells produce IFN-γ, which has been shown to restore HSV-induced MHC class I downregulation and upregulate MHC class II in infected keratinocytes (87). CD4 T cell depletion studies in mice provide evidence of the critical role of CD4 T cells in the immune response to HSV. For example, CD4 deficient or depleted mice fail to recruit CD8 T cells to the vaginal epithelium. CD4 T cell IFN-γ stimulates epithelial cells to secrete CXCL9 and CXCL10, which recruits CD8 T cells to the site of infection (88). In human studies, CD4 T cells have been observed persisting in genital skin at the site of HSV2 reactivation for at least 6 months post-healing (89) and continue to produce IFN-γ early after HSV antigen exposure and lesion healing (90). Similarly, in human recurrent herpetic lesions, CD8 T cells infiltrate later than CD4 T cells (52), and their recruitment into genital lesions is strongly correlated with viral clearance, confirmed by the selective depletion of CD4 T cells (48).

Upon lesion healing, HSV specific CD8 T cells persist at the dermo-epidermal junction adjacent to peripheral nerve endings in small, enriched clusters, and function as sentinels for reactivation in the female genital tract (91, 92). Resident HSV-specific CD8 T cells encounter HSV quite frequently, and as such express genes for antiviral function, chemotaxis, and recruitment (93), as well as a lack of chemokine receptor expression for egress and recirculation, and the ability to produce cytolytic granules during clinical quiescence (94). These findings demonstrate that these cells remain active in immunosurveillance after episode clearance. HSV-specific CD8 T_*RM*_ located in genital skin and mucosa have also been identified as CD8αα + T cells that express two CD8α chains, instead of an α and β chain. This homodimer expression has been associated with high affinity antiviral effector T cells (94).

Recently, studies of CD8 T cells and HSV have focused on investigating the spatial distribution of CD8 T_*RM*_ cells. Despite CD8 T_*RM*_ cells remaining in the genital tract as sentinels to protect against recurrences, shedding continues to occur and at variable rates between individuals. Schiffer and colleagues developed a mathematical model that spatially models the effects of variability of CD8 T_*RM*_ cells in HSV lesions, as well as HSV replication and spread. The model found that high levels of overall CD8 T cell density did not equate with total control of HSV and that high shedding drove frequent mucosal T cell turnover. HSV was also found to capitalize on the spatial heterogeneity of local immunity, exploiting the gaps and allowing reactivation to occur (95).

Schiffer and colleagues, using a mathematical model, found that HSV infection did not induce sufficient T_*RM*_ cells in the human genital tract to eliminate reactivation, and that strict spatial distribution is maintained during infection, as was found in murine models. The strict distribution and heterogeneity of T_*RM*_ cells provide areas for HSV replication to occur upon reactivation. The spatial distribution and heterogeneity of T_*RM*_ cells calculated from the mathematical model was also confirmed in histological genital biopsies. Understanding how genital tract T_*RM*_ cells are spaced in the tissue provides insight as to how reactivation continues to occur, even in their presence (96).

Therefore, CD8 T cells have been found to play important roles in HSV infection. They initially clear active lesions, then become T_*RM*_ cells, immune sentinels, that ensure reactivation is a rare occurrence. These studies on CD8 T cells suggest important insights into why previous vaccines, which have not been able to stimulate CD8 T cell activation, were unsuccessful. New vaccine designs should incorporate a focus on the stimulation of CD8 T cells and induction of a T_*RM*_ population that remain in the tissue as sentinels, ready for an encounter with HSV. Such vaccines would need to induce high T_*RM*_ cell numbers in the genital tract to overcome heterogeneous spatial distribution and provide higher killing efficiency and IFN-γ production (96).

Regulatory T cells (Tregs) are a population of CD4 T cells that suppress T cell effector functions. They are characterized by the expression of CD4, CD25, and the transcription factor Foxp3 (97). Tregs are an inherent component of any immunological response as they silence and suppress effector and cytotoxic immune responses to ensure harm does not come to the body. The role Tregs play in HSV lesions is controversial. Some murine studies have found that Tregs are beneficial either in facilitating an effective immune response or suppressing immunopathology. Tregs are essential for promoting the accumulation of HSV specific CD4 T cells in infected tissue and ensuring DCs traffic to the appropriate draining lymph node from the vaginal mucosa, resulting in effective CD4 T cell priming (98). However, other murine studies found that Tregs suppressed T cell effector responses to HSV. Depletion of Tregs before HSV infection significantly enhances HSV-specific CD8 T cell cytotoxicity in neonatal mice, and significantly enhances the IFN-γ responses of CD4 and CD8 T cells in both adult and neonatal mice (97). Furthermore, depletion of Tregs prior to HSV infection significantly decreases skin lesion severity and granulocyte cell numbers at the site of ganglionic spread from flank HSV2 (99). In human genital biopsies from HSV2 recurrent lesions, the density of Tregs directly correlated with HSV2 titers (100) Thus, it may be the balance between effector T cells and Tregs that determines whether Tregs are beneficial or detrimental during HSV infection.

A significant limitation of murine HSV infection models is that HSV does not cause recurrent lesions in mice, and so the role of Tregs in reactivation cannot be assessed. Therefore, it is important to assess the role of human Tregs in response to HSV infection. One study conducted on the peripheral blood of HSV+ patients found that CD4+CD25+ memory Tregs suppressed the proliferation of HSV specific CD4 T cells at times of clinical quiescence (101). It is known that high numbers of Tregs infiltrate the site of viral reactivation in genital skin biopsies and persist in proximity to T cells, specifically during reactivation. There is also a correlation between high Treg numbers and increased viral replication, indicating that Tregs may be suppressing immune effectors and allowing virus to proliferate. This correlates with the observation that Tregs were found to localize with CD4 T cells in the upper dermis (100). Shedding biopsies also had significantly higher ratios of Tregs to other T cells, and this affected the clinical presentation of disease; for example, an increase in Tregs could result in insufficient effector function (100). In the context of human HSV infection, the evidence suggests Tregs could be more detrimental than beneficial, particularly during virus reactivation. Therefore, an additional consideration for new vaccine designs could be the addition of adjuvants that suppress the activation of Treg responses, particularly in immunotherapeutic vaccines that aim to reduce virus reactivation.

Gamma-delta (γδ) T cells are non-conventional T cells that are uniquely defined by the expression of a γδ TCR, unlike conventional CD4 and CD8 T cells which express an αβ TCR. γδ T cells are enriched in epithelial tissues such as skin, where they maintain epidermal integrity (102). Studies in mice have investigated the role of γδ T cells in HSV infection. One study found that γδ T cells were protective, limiting the severity of HSV1 induced epithelial lesions and preventing the development of lethal viral encephalitis (103). They provided evidence that γδ T cells decrease viral replication and restrict viral progression into the brain. Another murine study found that epidermal γδ T cells were the first immune effector to encounter HSV and were directly infected, prior to the infection of Langerhans cells (104). However, human γδ T cells are different to their murine counterparts. Murine γδ T cells reside in both the epidermis and dermis, whereas human γδ T cells mainly reside in the dermis and near the dermo-epidermal junction (105). Some reports investigating human blood γδ T cells suggest that they could play a protective role in antiviral immunity, particularly to HSV (106, 107). However, the role of skin γδ T cells during HSV infection has not been investigated in human skin or genital mucosa and such studies could reveal important differences between the role of murine and human γδ T cells in HSV infection and whether they are important targets for vaccine design.

#### 2.2.4. The Role of Dendritic Cells in Stimulating HSV Immunity

Dendritic cells (DCs) are the most important bridge between the innate and adaptive immune system. They patrol blood and tissue compartments to detect pathogens and take up antigens, after which they mature and migrate to lymph nodes where they present the antigens to naïve T cells, thereby activating the adaptive immune response (108). Several subsets of DCs have been identified in various tissue compartments (e.g., blood, skin, liver, brain etc.).

Due to limitations in obtaining tissue-derived human DCs, earlier studies of the role and response of human DCs to HSV infection made use of model DCs generated from monocyte-derived DCs (MDDCs). These studies provided evidence that immature DCs could be productively infected by HSV, HSV induced apoptosis in human MDDCs (a process that HSV normally inhibits) and that uninfected DCs pulsed with apoptotic HSV-infected DCs could cross-present and stimulate HSV specific CD8 T cells (109, 110). These models still must be confirmed in tissue-derived human DCs; a complex process.

#### 2.2.5. The Complexity of the DC/Macrophage Network in Human Skin

It has been known for a long time that Langerhans cells (LCs) are the major DC subtype located in the epidermis. Whether LCs were important in HSV infection was first shown in the 1980s, where mice whose skin was abraded, leading to the fleeing of LCs, and inoculated with HSV1, had an increase in viral pathogenicity due to the absence of LCs. When LCs were present, interactions between LCs and HSV1 were observed as early as 2 h.p.i, and LCs became HSV1 gD positive, indicative of virus uptake (111).

A more recent study of LCs in mice following HSV infection showed that LCs became infected with HSV, however uninfected or bystander LCs were the main emigrant DC subset at 24 h.p.i. Additionally, most infected LCs failed to downregulate E-cadherin (preventing their emigration) and became apoptotic (104). When investigating the role of LCs in humans, one major difference is seen. LCs still become productively infected, mature and become apoptotic, however all infected LCs migrate into the dermis (112). Therefore, unlike murine LCs, where HSV infection seemed to inhibit migration of at least a significant proportion, in human LCs HSV infection induced migration to the dermis. Such differences highlight the importance of examining the immune response to HSV in human skin and in particular the role of subsets of human DCs.

In recent years, the development of technologies such as single cell RNA-sequencing have facilitated the classification of DC subsets. In human dermis, the two main DC subsets are conventional DC type 1 and 2 (cDC1 and cDC2) (113). cDC1s are a minor subset proportionally, but are highly efficient at cross-presentation of exogenous antigen to CD8 T cells (114). They are characterized by the expression of CD141, XCR1, the C-type lectin receptor CLEC9A and TLRs 1, 2, 3, 6, 7, and 8, and are recognized as the equivalent of murine CD103+/CD8α+ cross-presenting DCs (115–117). The major dermal DC subset are cDC2s, which have conventional antigen-presenting capacity to stimulate CD4 T cells, but also have some ability to cross-present to CD8 T cells (117, 118). They express CD1a, CD1c, CD11b, CD11c, and some express langerin (108) and may be the equivalent of murine submucosal CD11c+ and CD11b+ DCs. Single cell analysis has also complicated the definition of what is considered a DC or a macrophage. Dermal CD14+ mononuclear phagocytes (MNPs) were originally classified as DCs due to their ability migrate out of tissue explants, their expression of MHC class II and CD1c, and ability to influence T cells; all properties that dermal DCs have (119, 120). However, there is no known murine equivalent to human CD14+ DCs, yet mice and humans tend to have homologous cells (117, 121). Furthermore, a study found that CD14+ MNPs (which also express DC-SIGN) were transcriptionally and functionally similar to tissue resident monocyte derived macrophages (MDMs). For example their ability to stimulate memory T cells like macrophages but not stimulate naïve T cells, an ability unique to DCs. However, these cells also have DC properties in their ability to migrate out of tissue, making them a MDM with DC-like ability (122).

It has been known for some time that the process by which skin DCs take up HSV and present antigen to CD4 and CD8 T cells leading to the development of memory T cells is complex. Several studies have tried to unravel this complexity and define the process in murine models. It has been shown in mice that LCs take up HSV in the epidermis (111), but they do not present HSV antigen to T cells in lymph nodes. Neither LCs nor lymph node resident DCs present HSV2 antigens to CD4 T cells, but submucosal CD11c+ and CD11b+ DCs (cDC2s) do (123). Furthermore, naive CD8 T cells are primed by CD8α+ DCs and CD103+ dermal DCs (cDC1s) (124, 125) and the latter are the predominant cells transporting HSV antigens out of murine skin explants (104).

**Figure 1:**
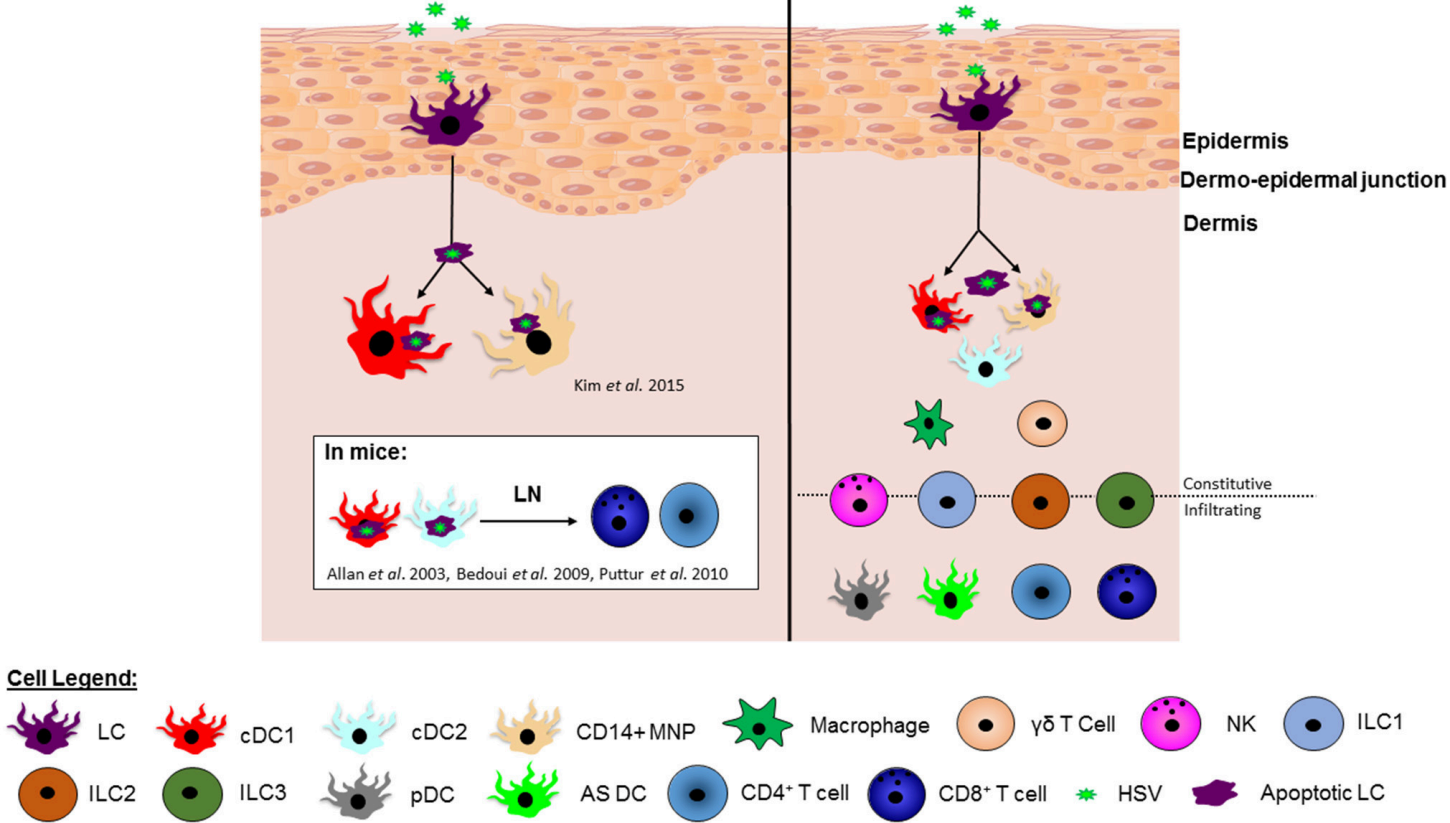
The HSV viral relay and localization of immune cell subsets in human skin. **(Left)** In humans, HSV infects Langerhans cells (LCs) causing them to mature and migrate to the dermis and undergo apoptosis. Once in the dermis, HSV infected apoptotic LCs have been observed in clusters with and taken up by dermal cDC1s and CD14^+^ MNPs (112), potentially for antigen presentation to T cells. In mice it is known that murine dermal cDC1s and cDC2s present HSV antigen to CD8^+^ and CD4^+^ T cells in the lymph node, however this has not yet been shown in human studies.**(Right)** Whilst we have pieced together multiple cellular players in this viral relay, there are an abundance of other innate and adaptive immune cells residing in the dermis, including additional DC subsets, macrophages and γδ T cells, as well as infiltrating immune cells, such as pDCs and T cells. NK cells and ILCs are found both constitutively in skin in low numbers and also infiltrate into the skin during infection or inflammation. There is increasing evidence that at least some of these additional cell types influence the developing immune response to HSV infection in the skin and further illuminating this complex picture would inform vaccine design.

In our recent human study, we investigated the interaction of HSV-infected LCs with dermal cDC1s in human inner foreskin explants and in biopsies of initial herpes simplex virus lesions. HSV1 infected LCs became apoptotic and migrated to the dermis to interact with cDC1s in clusters. LC fragments were detected within some cDC1s, and cDC1s emigrated from HSV1 infected explants, similar to CD103+ dermal DCs in murine models. Additionally, DC-SIGN+ MNPs were also observed in clusters interacting with HSV-infected LCs in the dermis (112). Therefore, this study demonstrated that epidermal LCs take up HSV, become infected and transfer the virus or viral antigens to subsets of dermal DCs/MNPs, facilitating viral relay. This has filled an important gap in knowledge of the immunological processes facilitating HSV antigen presentation to T cells. However, important questions remain: What role do human cDC2s play in interactions with HSV infected LCs? Are there differences in the interactions of different dermal DC/MNP subsets with the LCs that could determine their specific contributions to the activation of CD4 and CD8 T cells? By understanding the roles of specific human DC subsets in response to HSV infection, it should drive vaccine design toward stimulating pathways that induce the same immune responses as natural infection and, in particular, CD8 T cell responses that were not induced by previous vaccine candidates. A summary of the HSV viral relay and localization of immune cell subsets in human skin is shown in Figure 1.

## 3. Building on Knowledge of Natural Immunity to Design a Vaccine

### 3.1. Challenges of Designing a Protective Vaccine

Prophylactic and immunotherapeutic vaccines have different goals and as such there are challenges to overcome in the development of a successful prophylactic vaccine that are not critical for an immunotherapeutic vaccine. Since prophylactic vaccines aim to prevent acquisition of a pathogen, they need to stimulate effective primary immune responses at the site of pathogen entry. To generate primary immune responses, naïve T cells require two signals to differentiate into effector cells: an antigen-specific signal and a second costimulatory signal (such as CD80/86 ligation of CD27). DCs are the critical cell type for stimulating naïve T and B cells as they provide the second costimulatory signal to T cells that other “secondary” or “non-professional” antigen presenting cells cannot provide. Therefore, a successful prophylactic vaccine needs to stimulate the appropriate DCs. In contrast, immunotherapeutic vaccines aim to reduce morbidity by reducing clinical episodes, and reduce transmission by reducing viral shedding. This may be an easier immunological task than prophylaxis, as it relies on re-stimulating already existing memory T cell responses. Compared to stimulating naïve T cell responses, memory T cells are more abundant and do not require a costimulatory signal for activation. Therefore, a much wider range of immune cells than DCs can act as antigen presenting cells (including keratinocytes and monocytes). This also means memory T cell stimulation is more likely to occur in the periphery (91, 94). It is undetermined yet whether naïve HSV-specific T cell priming occurs in mucosal tissues or only in lymph nodes.

There is now compelling evidence that the presence of T_*RM*_ cells and neutralizing antibodies in the mucosa are critical for protection against release of virus from the DRG and also likely to prevent virus entry into the DRG during initial infection. T_*RM*_ cells may also be important in restricting reactivation in the DRG (86). It is therefore important to consider how to design a prophylactic vaccine that will induce the development of local T_*RM*_cells and mucosal antibody to prevent infection with HSV as recruitment of B and T cells from the blood may be too slow to prevent viral seeding of the nerves.

### 3.2. Targeting Key Antigens and Epitopes

HSV1 and 2 consist of double-stranded DNA contained in a capsid, surrounded by a tegument layer and an envelope containing glycoproteins including gB, gC, gD, and gH/gL (126). HSV replication involves the production of rounds of viral proteins for the assembly of the virus, beginning with immediate-early (IE), followed by early (E) then late (L) structural proteins (127). The immune response is capable of targeting many of these viral components and it is important that a vaccine stimulates responses to antigenic epitopes that have been identified as key targets for neutralizing antibodies, CD4 and CD8 T cells.

The late structural proteins gD and gB are dominant targets for HSV neutralizing antibodies, of which multiple epitopes are recognized (128, 129), along with gC and gH/L, specifically seen in human sera directed against HSV1 (19, 128–131). Other glycoproteins, such as gK, have only been investigated in murine models (132). Therefore, gD and gB were used as immunogens in the Chiron trial and gD combined with dMPL (AS04) used in the Simplirix and Herpevac trials. In the Herpevac trial, HSV2 gD was seen to confer protection for genital infection caused by HSV1 (but not HSV2), which correlated with high gD antibody titers, supporting the importance of antibodies in mediating this protection. When investigating the antibody response elicited from this vaccine in guinea pigs, the protection provided against genital disease was due mostly to neutralizing antibodies directed against gD, with various epitopes recognized, such as ID3, DL6, and MC14. The more epitopes the animals recognized, the better protected they were against genital disease. Upon investigating the epitope-specific antibody responses in women from the Herpevac trial, it was found that significantly fewer crucial gD epitopes were recognized compared to the guinea pigs (133). The recently developed trivalent vaccine candidate containing gC, gD, and gE provided sterilizing immunity in 98% of guinea pigs due to the high levels of plasma and mucosal neutralizing antibodies induced (69). Anti-gE is aimed at preventing cell to cell spread. Human antibody responses to this vaccine have not yet been assessed. Perhaps assessment of the efficacy of this vaccine and any future vaccines should evaluate epitope-specific antibody responses, such as to the gD2 epitopes ID3, DL6, and MC14.

Interestingly, a live attenuated viral vaccine, with a gD deletion, elicited mucosal antibodies with low neutralization activity but high antibody-dependent cellular cytotoxicity (ADCC) activity, provided sterilizing immunity in murine models and passively transferred immunity against vaginal infection with multiple clinical isolates (134–136). It is noteworthy that they authors did not include complement in their neutralization assays, which should be considered as an alternative mechanism to ADCC. The authors propose that the removal of the immunodominant gD protein may unmask alternative epitopes important in a protective immune response or remove a possible immunosuppressive effect of gD. Furthermore, another live, attenuated vaccine candidate HSV529 (deleted for UL5 and UL29) was shown to induce significant HSV2-specific antibody dependent ADCC, as well as neutralizing antibodies, in humans. (137). ADCC activity may warrant further attention in vaccine evaluations.

Although the above studies provide evidence for the importance of vaccines eliciting strong neutralizing antibody responses, many of the previous human clinical trial vaccines did induce neutralizing antibodies and yet were unsuccessful (138, 139). Although it has been suggested that this may be partially explained by a lack of epitope-specific responses, also neutralizing antibodies may not be sufficient by themselves to provide protection against HSV infection. CD4 and CD8 T cells are also likely to be required. Therefore, the ability to stimulate them in a vaccine needs to be improved from the previous vaccine candidates.

CD4 and CD8 T cells respond to various viral proteins, some of which overlap with those that neutralizing antibodies recognize. CD4 T cells predominately respond to late HSV glycoproteins, such as gD, gB, gC, and gH and the tegument protein VP16 (19, 87, 140). Several immunodominant HSV2 gD epitopes are recognized by CD4 T cells from both HSV1 and HSV2 seropositive patients (141), and such cross-reactive epitopes for HSV1 and HSV2 would be advantageous to use in a vaccine to target both viruses at the same time. CD4 T cells can also recognize the tegument protein UL49 and capsid protein VP5 (140). CD8 T cells recognize a variety of HSV proteins, especially IE and E viral proteins such as ICP27, ICP4, and ICP0 (87, 142), as well as several tegument and capsid proteins (143). Recent studies have found a conserved epitope between VZV, HSV, and EBV that is recognized by CD8 T cells, that has now extended to 13 conserved epitopes between VZV and HSV that are recognized by both CD4 and CD8 T cells (144, 145). Finally, a study has found that gD is selectively taken up by cDC1s, which can then cross-present to CD8 T cells, meaning that gD may be an important target for both CD4 and CD8 T cells (146), as well as B cells. The identification and use of conserved and cross-reactive epitopes in new vaccine designs may lead to the possibility of targeting multiple immune cells against multiple herpesviruses in the one vaccine.

Many studies that have identified HSV-specific T cell epitopes in humans have investigated the responses of T cells derived from PBMC. However, recent evidence indicates that there may be compartmentalization of T-cell receptor (TCR) repertoires and expansion of particular T cell clones at distinct anatomical sites. This may have important implications for how T cell responses to potential vaccine candidates should be assessed. One study compared the frequency of cervical and PBMC-derived HSV2-reactive CD4 T cells in HSV2 infected women and found there was a 25-fold enrichment of cervical HSV2 reactive CD4 T cells compared to PBMC, demonstrating that there are differences in frequency of HSV-specific CD4 T cells at different anatomical sites in natural HSV2 infection (92). Furthermore, recent data from the same lab presented at the International Herpesvirus Workshop investigated the overlap of TCR sequences between genital skin and PBMCs in HSV2 infected patients and also in response to an immunotherapeutic vaccine. The data suggest there is very little overlap in the TCR repertoires of tissue resident T cells in genital skin and those found in PBMCs. Therefore, it will be important to evaluate tissue-based immune responses in response to vaccines (147, 148).

### 3.3. Vaccine Delivery

As HSV infects the genital mucosa, vaccine strategies need to be effective at developing protective immunity at mucosal surfaces. One such strategy is direct immunization of the genital tract, a strategy that has been successful in animal models. A group investigating a live attenuated, replication defective HSV2 vaccine candidate (HSV2-gD27) has shown that intravaginal delivery gave the best protection against HSV2 intravaginal challenge compared to intranasal, subcutaneous, or intramuscular delivery (149). However, intravaginal vaccination may be a difficult or impractical strategy to use in human trials. As an alternative approach, Shin and Iwasaki developed the “prime and pull”strategy where systemic T cells were primed by parenteral vaccination then pulled to the genital mucosa by the topical application of CXCL9 and CXCL10 (150). Long term CD8 T_*RM*_ cells were established in mice, which conferred protection against HSV2 challenge via IFN-γ production (151).

Experimental vaccines using nanoemulsion-based adjuvants are also being investigated for their efficacy in generating mucosal immunity. Intranasally administered nanoemulsiion vaccines have demonstrated the induction of high antibody titers, robust Th1-skewed T cell responses and potent Th17 responses in RSV and TB vaccines (152, 153). A nanoemulsion vaccine for HSV2 is also being developed by BlueWillow Biologics (formerly known as NanoBio Corporation). Preliminary evidence indicates that the intranasal vaccine can protect naïve animals from acute genital HSV2 infection and the establishment of latency, and also significantly reduces lesion recurrence in already infected animals (154). It would be worthwhile to further investigate whether intranasally administered nanoemulsion vaccines generate protective systemic and mucosal HSV immunity without directly immunizing the genital tract. Other experimental vaccines being investigated include the use of peptides as the epitope, either lipopeptides or synthetically designed peptides, or the use of nanoparticle adjuvants. None of these experimental vaccines are currently in Phase I clinical trials but they do hold some promise. Peptide based vaccines are the most developed and promising with these vaccines able to stimulate high titers of polyfunctional cytotoxic CD8 T cells that are found both locally in the genital mucosa and draining lymph nodes, as well as systemically. These CD8 T cells also induced high levels of IFN-γ, IL2, IL12, and TNF, and protected against lethal rechallenge of HSV (155, 156). Work on nanoparticle adjuvants is limited but work on a calcium phosphate based nanoparticle and HSV2 epitope was shown to lead to enhance mucosal and systemic protection. This vaccine was shown to protect against lethal rechallenge with live virus, as well as induce specific IgG and IgA responses. However, no adaptive response was induced by this vaccine (157).

### 3.4. Vaccine Adjuvants

In contrast to live attenuated vaccines, recombinant protein vaccines are often formulated with an adjuvant to act as antigen carriers (Eg. alum, emulsions such as MF59, liposomes) and as immune stimulants (namely TLR agonists), often combined as “adjuvant systems.” Adjuvants can modulate the immune response by activating DCs (replacing endogenous pathogen stimuli), and stimulate the appropriate immune pathway via different patterns of cytokine production. With an expanding pool of chemically well-defined and functionally characterized adjuvants available, there is an opportunity to tune the immune response to the desired outcome.

A protective recombinant protein vaccine will need to induce a combination of robust neutralizing antibody, CD4 and CD8 T cell responses and facilitate the establishment of T_*RM*_ cells. A number of adjuvants have been shown to induce neutralizing antibody responses including the traditionally used alum, MF59 which was used in the Chiron subunit vaccine (18), dMPL which was used in the Simplirix vaccine (20, 21), and more recently the combination of CpG and alum was used in the recent trivalent vaccine containing HSV2 gC, gD and gE. Notably, although this vaccine was administered intramuscularly in animal models, it elicited mucosal neutralizing antibodies that were protective upon intravaginal challenge (69).

However, for the activation and polarization of T cell responses, there are striking differences in the type of responses stimulated by different adjuvants. Alum adjuvanted vaccines do not elicit strong T cell responses (158, 159). Adjuvants such as MF59 and ISCOMs, as well as TLR2 and TLR5 ligands, enhance T cell responses without altering their Th1/Th2 balance of responses. In contrast, more polarized Th1 cell responses are elicited by adjuvants that incorporate agonists of TLR3, TLR4, TLR7-TLR8, and TLR9. Complete Freund's adjuvant (CFA) and CAF01 induce mixed Th1 and Th17 cell responses. Thus, selection of an appropriate adjuvant is influenced by the type of CD4+ T cell response required for protection.

Simplirix was the first partially successful HSV vaccine and this was attributable to the Th1 pattern of cytokines (IFN-γ) induced by the adjuvant dMPL, however no CD8 T cell responses were detected. One of the main hurdles in the advancement of vaccine development has been finding adjuvants that enhances cross-presentation, which is necessary for the induction of CD8 T cell responses to soluble antigen. Saponin-based adjuvants have been shown to induce strong T cell responses and in particular memory CD8 T cell responses, and their use in recently trialled immunotherapeutic vaccines has shown some success. The highly successful RZV vaccine for herpes zoster contains dMPL formulated together with QS21, a saponin, in liposomes. RZV induced VZV-specific CD4 T cells as well as memory CD8 T cells, although not naive CD8 T cells (15). Similarly, the Agenus HerpV vaccine contains a patented QS21 stimulon adjuvant and the Genocea vaccine contains a saponin Matrix M2 adjuvant. Both the immunotherapeutic Agenus and Genocea vaccines induced a combination of neutralizing antibody, CD4 and CD8 T cell responses in animal models. In the human clinical trial of the Genocea vaccine, equivalent CD8 T cell responses were induced to both HSV gD and ICP4, confirming that gD contains CD8 T cell epitopes, and that saponin-based adjuvants are able to induce memory CD8 T cell responses through cross presentation (160, 161).

In order to achieve the breadth of immune responses required (antibody, CD4 and CD8 T cells) in a vaccine for HSV, it may also be important to consider targeting adjuvants to additional immune cells that may assist in enhancing the overall responses. For example in our previous study of the LC-dermal DC viral relay, we suggested that for the targeting of dermal DC subsets by subunit vaccines, adjuvants may need to simulate the immune effects of HSV infected apoptotic LCs (112). Additionally, cell types that have traditionally been overlooked in the design of vaccine candidates, such as NK cells, should also be considered as targets for vaccine adjuvants, especially since NK cells are known to mature DCs and augment CD4 T cell responses (49). NK cells may perhaps also augment CD8 T cell responses as it appears NK cells can stimulate cross-presenting DCs (162, 163).

It is also important to consider whether adjuvants can be used to suppress certain aspects of the immune response that may not be beneficial for an effective response to HSV, such as Tregs. As Tregs are a component of any immune response, they are likely to be recruited in the context of vaccination. A recent study focusing on T cell vaccines for influenza, found that primary and repeated vaccination with viral peptides alone induced antigen specific FoxP3+ Tregs, but that the addition of certain adjuvants, such as CpG, suppressed this phenomenon. This study also found that in the context of influenza, depletion of vaccine induced antigen specific Tregs promoted viral clearance, indicating that Tregs have an inhibitory role *in vivo* (164). Most studies investigating Tregs in the context of HSV vaccination have used mouse models, where Tregs were found to be beneficial (98, 165), however the trend is not carried over into humans. Although not specifically studied in vaccines, Tregs have been shown to decrease effector T cell function in HSV infection as discussed previously (100, 101). Therefore, it is possible that adjuvant suppression of Tregs could be beneficial for a HSV vaccine, and that this is not an influenza specific phenomenon. However, it is also possible that Treg suppression could cause increased inflammation in response to the vaccine and in response to HSV infection. Therefore, the suppression of Tregs would need to be tested to determine whether it is ultimately beneficial or harmful in the context of HSV vaccination.

It is important to note that there are some concerns about potential safety issues in manipulating the immune response with adjuvants e.g., the possibility of inducing or reactivating autoimmune disease. So far, in tens of thousands of subjects immunized with RZV this has not been observed. However, extensive post-marketing surveillance will be required. Furthermore, the RZV adjuvant QS21 has been shown to elicit a high degree of systemic and local (infection site) reactogenicity as well as efficacy. Efficacy does not necessarily correlate with reactogenicity for individual subjects. However, whether the toxic and immunogenic aspects of such adjuvants can be dissociated, leading to chemical modifications, depends on a detailed understanding of the immunologic mechanisms of each. A summary of the developmental status of current HSV vaccine candidates is provided in Table 1.

**Table 1:** The developmental status of HSV vaccine candidates.

**Vaccine candidate**	**Company**	**Vaccine constitution**	**Developmental stage**	**References**
**SUBUNIT/S + ADJUVANTS**				
Simplirix/ Herpevac	GlaxoSmithKline	gD2 and AS04 (dMPL)	Ceased after Phase III trials	(21, 166)
GEN-003	Genocea	gD2 and Matrix M2	Ceased after Phase II trials	(167–169)
HerpV	Agenus	Peptide vaccine + QS-21 Stimulon	No development since Phase II trials	(170, 171)
VCL-HB01	Vical	gD2 +/- UL46 and Vaxfectin DNA vaccine	Ceased after Phase II trials	(172, 173)
COR-1	Admedus	gD2 codon optimized DNA vaccine	Phase IIb planned	(174–176)
NE-HSV2	BlueWillow Biologics	Nanoemulsion with gB2 and gD2 antigens	Pre-clinical, clinical trial planned	(154, 177)
HSV2 trivalent vaccine	University of Pennsylvania	gC2, gD2, gE2	Pre-clinical	(68, 178)
G103	Immune Design	HSV2 gD, UL19 and UL25	Pre-clinical	(179)
**LIVE-ATTENUATED**				
HSV529	Sanofi Pasteur	Replication defective HSV2, UL5, UL29 deletion	Phase I trial ongoing	(17, 180)
RVX201	Rational Vaccines	HSV2 ICP0 deletion mutant	Phase Ib/IIa planned	(181)
VC2	Louisiana State University	HSV1 with mutations in gK and UL20	Pre-clinical	(132, 165, 182)
R2	Thyreos LLC	HSV1 with UL37 R2 region mutation	Pre-clinical	(183)
HSV2 ΔgD2	Albert Einstein College of Medicine	HSV2 with US6 (gD) deletion	Pre-clinical	(134, 136)

## 4. Concluding Remarks

A new generation of vaccines aim to specifically manipulate the immune response or alternatively attenuate live vaccine candidates through specific mutations. Surprisingly, RZV has a higher degree of efficacy (and also more reactogenicity) than the live attenuated HZ vaccine, Zostavax. RZV is also more immunogenic (26, 27). Whether such higher adjuvant induced efficacy can be extended to vaccines against initial genital herpes infection remains to be proven. More antigens may be needed. These studies demonstrate that the need for a much more detailed understanding of initial protective immune responses and also the need to further analyse partially successful vaccines for immunologic correlates of efficacy (in protected vs unprotected patients) e.g., Genocea, Herpevac.

There remains much to be explored including the role of the microbiome in interacting with mucosal immunity. In sub-Saharan Africa, many women have a “diverse” vaginal microbiome without Lactobacilli which increases the likelihood of HIV and possibly HSV acquisition (86, 184–187). How mucosal immunity is altered and how this might be improved by immunization for HSV are topics for future investigation.

Thus, a successful prophylactic vaccine against initial genital herpes will need to prevent seeding of the neuronal ganglia by both HSV1 and HSV2. In addition to inducing high levels of neutralizing antibodies which are known to penetrate the epidermis, the vaccine would probably need to induce resident immune cells that can quickly migrate into the stratified squamous epidermis or produce rapidly diffusing protective cytokines upon infection and contain/destroy the virus before it enters nerve terminals in the skin. We now know that even if viruses such as HIV obtain a “toehold” in mucosal epidermal cells they can be contained by these mechanisms. More needs to be known about the interaction of key innate and adaptive immune responses. It is becoming clear that multiple innate immune cells such as multiple DC subsets, NK cells, monocytes/macrophages and γδ T cells are interacting in the mucosae during initial HSV infection and together with antibody and T cells may all have a role in successful control or protection of initial infection.

## Author Contributions

NT and JS are equal first authors. AC is the corresponding author. NT, JS, KS, and AC all contributed to writing and editing the manuscript.

### Conflict of Interest Statement

AC reports other funding to his institution from GSK, Merck and BioCSL/Sequiris, outside the submitted work. The remaining authors declare that the research was conducted in the absence of any commercial or financial relationships that could be construed as a potential conflict of interest.

## Abbreviations

HSV1/2: Herpes Simplex Virus 1/2
HIV: Human Immunodeficiency Virus
VZV: Varicella Zoster Virus
DC: Dendritic Cell
dMPL: deacylated Monophosphoryl Lipid A
MHC: Major Histocompatibility Complex
IFN: Interferon
pDC: Plasmacytoid Dendritic Cell
NK: Natural Killer Cell
AS DC: Axl^+^ Siglec6^+^ Dendritic Cell
ILC: Innate Lymphoid Cell
DRG: Dorsal Root Ganglia
T_*RM*_: Tissue Resident Memory T cell
Tregs: Regulatory T Cells
LCs: Langerhans cells
MNPs: Mononuclear Phagocytes
PBMCs: Peripheral Blood Mononuclear Cells
